# Epstein-Barr Virus (EBV) Latent Protein EBNA3A Directly Targets and Silences the *STK39* Gene in B Cells Infected by EBV

**DOI:** 10.1128/JVI.01918-17

**Published:** 2018-03-14

**Authors:** Quentin Bazot, Kostas Paschos, Martin J. Allday

**Affiliations:** a Molecular Virology, Department of Medicine, Imperial College London, London, United Kingdom; University of California, Irvine

**Keywords:** EBNA3, Epstein-Barr virus, transcriptional regulation, virology

## Abstract

Epstein-Barr virus (EBV) establishes latent infection in human B cells and is associated with a wide range of cancers. The EBV nuclear antigen 3 (EBNA3) family proteins are critical for B cell transformation and function as transcriptional regulators. It is well established that EBNA3A and EBNA3C cooperate in the regulation of cellular genes. Here, we demonstrate that the gene *STK39* is repressed only by EBNA3A. This is the first example of a gene regulated only by EBNA3A in EBV-transformed lymphoblastoid cell lines (LCLs) without the help of EBNA3C. This was demonstrated using a variety of LCLs carrying either knockout, revertant, or conditional EBNA3 recombinants. Investigating the kinetics of EBNA3A-mediated changes in *STK39* expression showed that *STK39* becomes derepressed quickly after EBNA3A inactivation. This derepression is reversible as EBNA3A reactivation represses *STK39* in the same cells expressing a conditional EBNA3A. *STK39* is silenced shortly after primary B cell infection by EBV, and no *STK39*-encoded protein (SPAK) is detected 3 weeks postinfection. Chromatin immunoprecipitation (ChIP) analysis indicates that EBNA3A directly binds to a regulatory region downstream of the *STK39* transcription start site. For the first time, we demonstrated that the polycomb repressive complex 2 with the deposition of the repressive mark H3K27me3 is not only important for the maintenance of an EBNA3A target gene (*STK39*) but is also essential for the initial establishment of its silencing. Finally, we showed that DNA methyltransferases are involved in the EBNA3A-mediated repression of *STK39*.

**IMPORTANCE** EBV is well known for its ability to transform B lymphocytes to continuously proliferating lymphoblastoid cell lines. This is achieved in part by the reprogramming of cellular gene transcription by EBV transcription factors, including the EBNA3 proteins that play a crucial role in this process. In the present study, we found that EBNA3A epigenetically silences *STK39*. This is the first gene where EBNA3A has been found to exert its repressive role by itself, without needing its coregulators EBNA3B and EBNA3C. Furthermore, we demonstrated that the polycomb repressor complex is essential for EBNA3A-mediated repression of *STK39*. Findings in this study provide new insights into the regulation of cellular genes by the transcription factor EBNA3A.

## INTRODUCTION

Epstein-Barr virus (EBV) is a human DNA virus that belongs to the gammaherpesvirus family and that persistently infects >90% of the human population. Primary infection is usually asymptomatic when it occurs in childhood but can result in the benign lymphoproliferative syndrome known as infectious mononucleosis when it occurs later in life. After the primary infection, EBV persists in a latent state in memory B cells for the lifetime of infected individuals with intermittent viral production occurring in the oropharynx. Infection with EBV has etiologically been associated with several types of human cancer, including Burkitt lymphoma, Hodgkin lymphoma, peripheral natural killer/T-cell lymphoma, and nasopharyngeal and gastric carcinoma (1, 2). *In vivo*, early after infection, all EBV latency-associated genes are expressed, producing six EBV nuclear antigens (EBNA1, -2, -3A, -3B, and -3C and leader protein [LP]), three latent membrane proteins (LMP1, -2A, and -2B), two small noncoding RNAs (EBER1 and -2), and microRNA transcripts from the BamHI A region (BARTs) (2–5). These latency-associated gene products act together to activate the quiescent B cells into proliferating B blasts. In immunocompetent individuals, this proliferation of the infected B cells is controlled by the action of EBV-specific cytotoxic T lymphocytes that recognize and destroy the proliferating B blasts and is also controlled by signals leading B cells to differentiate into the resting memory state via the germinal center (GC) reaction (6–8). *In vitro*, however, EBV has the unique capacity to infect, activate, and induce the continuous proliferation (also known as transformation or immortalization) of quiescent B cells, leading to the establishment of lymphoblastoid cell lines (LCLs).

EBNA3A, EBNA3B, and EBNA3C are a family of three EBV nuclear antigens expressed from three genes arranged in tandem within a complex transcription unit functional only in B cells (reviewed in reference 9). Early genetic studies initially established that EBNA3A and EBNA3C are both required and essential for B cell transformation *in vitro* whereas EBNA3B is not necessary. All three proteins are well established as transcription factors that cooperate in the regulation of expression of thousands of host genes (10). However, it seems that they bind DNA not directly but via contacts with cellular cofactors and are largely targeted to chromatin at gene promoters and/or distal regulatory elements (11–16). The EBNA3-mediated regulation of transcription often involves long-distance chromatin interactions (chromosome “looping”) between promoter and enhancer elements mediated by EBNA3 proteins (reviewed in reference 17).

Among the different epigenetic silencing mechanisms, trimethylation of lysine 27 at histone 3 (H3K27me3) and DNA methylation play critical roles in the development of cancer and have been recently shown to be linked (18–21). H3K27me3 is catalyzed by the methyltransferase enhancer of Zeste homologue 2 (EZH2), a member and catalytic subunit of the polycomb repressor complex 2 (PRC2) (22, 23). This specific trimethylation is therefore considered a hallmark of PRC2-mediated repression leading to silencing of the target genes. DNA methylation is an epigenetic modification that maintains gene silencing with the addition of methyl groups to the fifth carbon position of the cytosine residues by DNA methyltransferases (DNMTs) (24).

EBNA3A and EBNA3C are known to epigenetically downregulate cellular genes involved in the regulation of the cell cycle and apoptosis such as *BCL2L11* (encoding the proapoptotic, BH3-only protein BIM) and the cyclin-dependent kinase inhibitors (CDKIs) p16^INK4a^ and p15^INK4b^ (25–30). Interestingly, it has recently been demonstrated that EBNA3A and EBNA3C block plasma cell differentiation in EBV-activated B cells by repressing the cyclin-dependent kinase inhibitor p18^INK4c^ and the transcription factor BLIMP-1 (31). The epigenetic mark H3K27me3 has always been associated with genes transcriptionally repressed by the cooperation of EBNA3A and -3C, and DNA methylation has been observed previously at the EBNA3A and -3C-coregulated gene *BCL2L11* (9). This extensive cooperation between EBNA3A and EBNA3C in gene regulation is well documented, and all genes identified to be regulated by EBNA3A have also been shown to be regulated by its coregulator EBNA3C, raising the question of whether or not EBNA3A can regulate genes on its own. Interestingly, we recently identified a cellular gene—*COBLL1*—regulated by only EBNA3C. However, a cellular gene regulated by EBNA3A alone remained to be identified.

Here—following leads from a microarray analysis performed using the recently established EBNA3A-ERT2 conditional LCLs (32)—we identified *STK39* as being a cellular gene repressed by EBNA3A. Using a comprehensive set of cell lines conditional for or having deletion of each EBNA3 protein, we were able to show that only EBNA3A is involved in *STK39* silencing in LCLs. Chromatin immunoprecipitation sequencing (ChIP-seq) and ChIP-quantitative PCR (qPCR) data were consistent with EBNA3A binding to a regulatory element and directly epigenetically repressing the *STK39* gene. Further characterization of the molecular mechanism by which EBNA3A silences *STK39* revealed a dependence on polycomb repressor complex 2 (PRC2) with the deposition of H3K27me3 as well as implication of DNA methyltransferases.

## RESULTS

### EBNA3A is the only EBNA3 protein that regulates the serine/threonine kinase gene *STK39*.

The EBNA3A protein has been shown to play a role in the regulation of cell survival in B cells immortalized by EBV (29, 30). To identify direct EBNA3A target genes involved in this cellular process, we used LCLs conditional for EBNA3A function (EBNA3A-ERT2 lines [32]), where the EBNA3A protein is functional only in the presence of the activating ligand for modified-estrogen receptor (4-hydroxytamoxifen [HT]) in the culture medium. We performed Affymetrix gene expression profiling using the 3A-ERT2 LCL cultured for 28 days with or without HT. Several well-known EBNA3A target cellular genes were found in the top 15 genes repressed by EBNA3A (Table 1) (29, 33, 34). One cellular gene, *STK39*, was of particular interest because it has recently been reported in the literature to have a role in apoptosis activation in B cell lymphoma. Interestingly, *STK39* was also found to be an EBNA3A target gene by Hertle and colleagues in a microarray analysis using LCLs where EBNA3A had been knocked out (EBNA3A-KO) (29).

**TABLE 1:** Top 15 cellular genes repressed by EBNA3A

Gene symbol	Fold change (HT vs washed)
CXCL9	−5.74
RGS13	−3.06415
ADAMDEC1	−2.44095
CLIC2	−2.31221
PCDH11Y	−2.29114
PRSS1	−2.28726
CXCL10	−2.25629
OR10G8	−2.125
TOM1L2	−2.11133
HIST3H3	−2.094
STK39	−2.06927
ADAM28	−2.06313
LSM14B	−2.04608
KRTAP6-3	−2.0368
C21orf99	−2.02141

**FIG 1:**
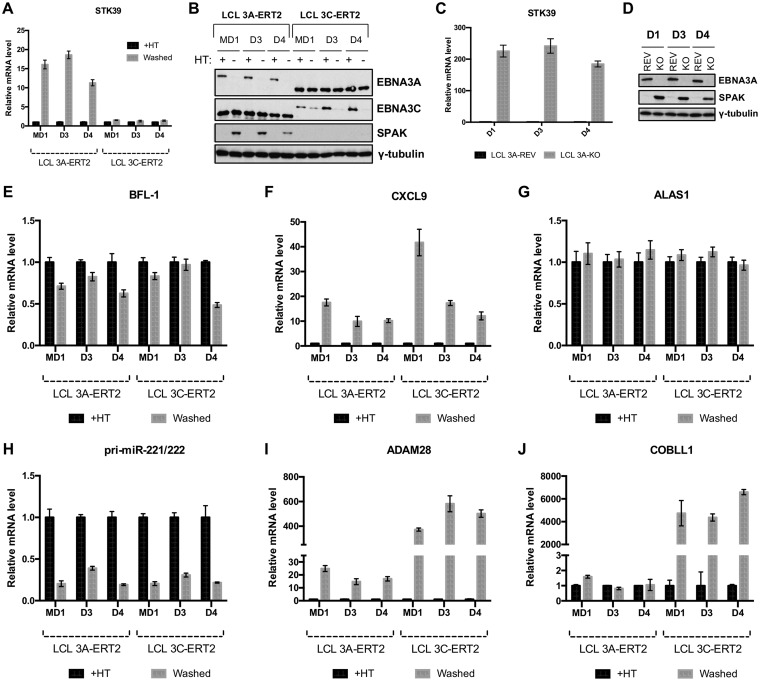
EBNA3A is required to repress the *STK39* gene. (A) *STK39* mRNA expression in three independent EBNA3A-ERT2 LCLs as well as three independent EBNA3C-ERT2 LCLs (MD1, D3, and D4) cultured for 28 days with (+HT) or without (Washed) HT. *STK39* gene expression was normalized to the endogenous control *GNB2L1* and is shown relative to LCL 3A-ERT2 MD1 (+HT), the STK39 level of which was set to 1. (B) EBNA3A, EBNA3C, SPAK, and γ-tubulin protein expression in EBNA3A-ERT2 and EBNA3C-ERT2 LCLs used in the experiment in panel A. (C) *STK39* mRNA expression in three independent EBNA3A-KO and EBNA3A-REV LCLs (D1, D3, and D4). *STK39* gene expression was normalized to the endogenous control *GNB2L1* and is shown relative to LCL 3A-REV D1, the *STK39* level of which was set to 1. (D) EBNA3A, SPAK, and γ-tubulin protein expression in EBNA3A-KO and EBNA3A-REV LCLs used in the experiment in panel C. (E to J) Expression levels of BFL-1 (E), CXCL9 (F), ALAS1 (G), pri-miR-221/222 (H), ADAM28 (I), and COBLL1 (J) were determined in 3 independent 3A-ERT2 LCLs and 3C-ERT2 LCLs used in the experiment in panel A.

In order to validate whether EBNA3A regulates *STK39* expression in the LCL 3A-ERT2 system, mRNAs from LCL 3A-ERT2 established from one mixed donor population of B cells (MD1) and two independent donor backgrounds (D3 and D4) were analyzed by reverse transcription (RT)-qPCR ∼30 days after HT removal (washed) or continuously cultured in HT (+HT) (Fig. 1A). Consistently, on removal of HT, STK39 mRNA greatly increased in all 3A-ERT2 LCLs. The level of SPAK (protein encoded by the *STK39* gene) was also investigated by Western blotting (Fig. 1B). With HT in the culture medium, the SPAK level was undetectable in LCL 3A-ERT2. In contrast, when HT was washed from the culture medium (−HT), the SPAK level was raised (Fig. 1B). Using three independent donor EBNA3A-KO LCLs and three LCLs established with revertant (REV) viruses (and therefore expressing all the latency-associated EBV proteins), we also found that when EBNA3A was deleted, there was a robust activation of the *STK39* gene that could be seen at both the RNA and protein levels (Fig. 1C and D).

Because members of the EBNA3 family cooperate extensively in the regulation of many cellular genes (10), it was important to determine whether EBNA3C and/or EBNA3B was involved in *STK39* regulation in LCLs. Using three LCLs conditional for EBNA3C function (LCL 3C-ERT2) established in the same donor background as the LCL 3A-ERT2, removal of HT for ∼30 days had no effect on STK39 mRNA level or SPAK protein (Fig. 1A and B). This is the first time that a cellular gene has been shown to be regulated by only EBNA3A, as so far, all the well-studied EBNA3A-regulated genes are also coregulated by EBNA3C (34–36). Very recently, *BFL-1* has been shown to be activated by EBNA3A in LCL 3A-ERT2 (30). However, this activation is not specific to EBNA3A, as the BFL-1 mRNA level is also regulated the same way in EBNA3C conditional cell lines (Fig. 1E). This is the same for CXCL9, which has been shown to be repressed by EBNA3A but is also repressed by EBNA3C (Fig. 1F). Control RNA *ALAS1* was unaffected by the EBNA3A or EBNA3C status of the LCLs (Fig. 1G), and expression levels of genes previously reported to be either activated (*pri-miR-221/222* [32]) or repressed (*ADAM28* [29, 33, 35]) by both EBNA3A and EBNA3C and of a gene regulated by only EBNA3C (*COBLL1* [11]) were as expected (Fig. 1H to J).

**FIG 2:**
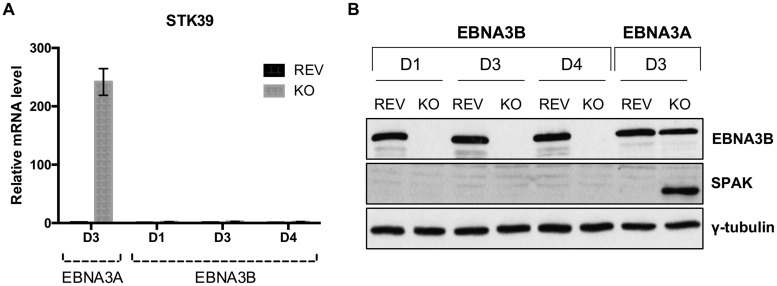
EBNA3B does not regulate *STK39* expression. (A) STK39 mRNA expression for LCL EBNA3A-KO and EBNA3A-REV D3 and three independent EBNA3B-KO and EBNA3B-REV LCLs (D1, D3, and D4). (B) EBNA3B, SPAK, and γ-tubulin protein expression in LCLs used in the experiment in panel A.

Similarly, using three independent EBNA3B-KO LCLs and three LCLs established with revertant viruses, it appeared that EBNA3B did not influence the regulation of the *STK39* gene (Fig. 2A and B). Taken together, these results demonstrate that the only EBNA3 protein needed for *STK39* silencing is EBNA3A.

### Kinetics of *STK39* repression by EBNA3A in LCLs.

**FIG 3:**
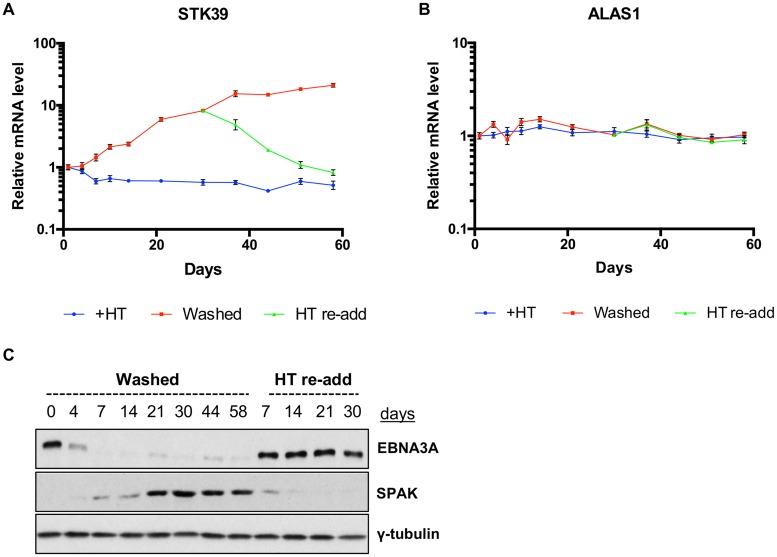
Kinetics of *STK39* derepression in EBNA3A conditional cell line. (A and B) Time course using EBNA3A-conditional LCL 3A-ERT2 MD1. Cells were grown over 60 days either in the presence of HT (+HT), in the absence of HT (washed), or with HT readded after 30 days in the washed state (HT re-add). Gene expression for *STK39* (A) and *ALAS1* (B) was normalized to the endogenous control *GNB2L1* and is shown relative to +HT at day 0. Data are representative of two independent time course experiments. (C) EBNA3A, SPAK, and γ-tubulin protein expression during LCL 3A-ERT2 MD1 time course in the absence of HT (washed) or after readdition of HT after 30 days in the washed state (HT re-add).

The genes regulated by the EBNA3 proteins are either epigenetically repressed or activated, and most EBNA3 target genes (e.g., *COBLL1* and *AICDA* for EBNA3C or *BIM/BCL2L11* for EBNA3A and EBNA3C) were shown to be reversibly regulated if either EBNA3A or EBNA3C was inactivated. However, we recently demonstrated that this is not always the case, as the repression of *p18^INK4c^* and *BLIMP-1* initiated by EBNA3A and EBNA3C is irreversible under the same conditions (31). Using the LCLs conditional for EBNA3A function (LCL 3A-ERT2 [Fig. 1A and B]), we have already shown that the *STK39* repression initiated by EBNA3A was reversed 30 days after removal of HT. We then investigated the kinetics of this derepression. To do that, we used a time course experiment where cell samples were harvested every 3 to 4 days for analysis (Fig. 3). LCL 3A-ERT2 cells were first either left cultured with HT or washed in order to remove the HT from the culture medium for a period of 30 days. Interestingly, the derepression of STK39 mRNA appeared quickly after inactivation of EBNA3A (7 to 10 days after removal of HT [Fig. 3A]) and continued to rise after the initial HT removal. Relative expression of the control housekeeping gene *ALAS1* was unaffected by the inactivation/reactivation of EBNA3A during the entire time course experiment (Fig. 3B). The regulation of *STK39* by EBNA3A could also be observed at the protein level (SPAK level [Fig. 3C]). SPAK level appeared quickly (7 days after the initial removal of HT) and mimicked the level of STK39 mRNA (Fig. 3A). After 30 days without HT, the activating ligand was readded into the cultured medium for another period of 30 days to investigate whether the reactivation of EBNA3A could quickly silence *STK39*. Interestingly, upon readdition of HT, the STK39 mRNA level significantly decreased and reached its initial level 3 weeks after reactivation of EBNA3A (Fig. 3A). The SPAK level was barely detectable 2 weeks after reactivation of EBNA3A and was completely undetectable thereafter (Fig. 3C).

### EBNA3A protein silences *STK39* by 15 days after EBV infection.

**FIG 4:**
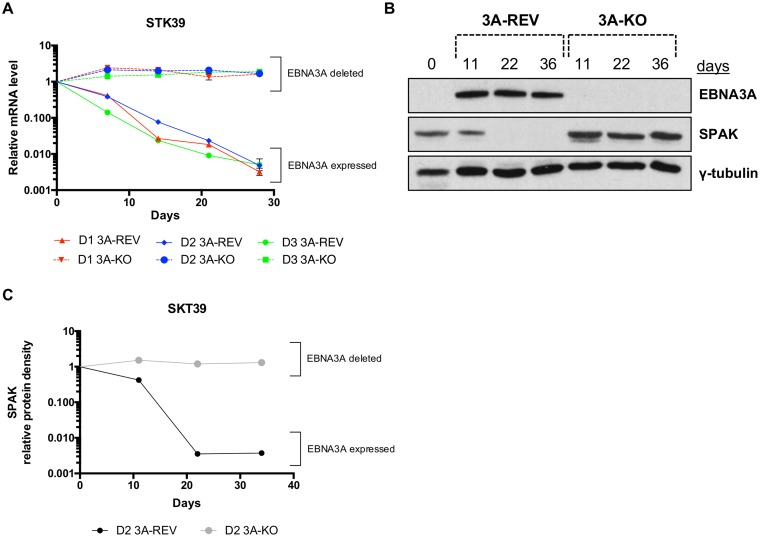
Repression of *STK39* after infection of primary B cells with EBV. (A) CD19^+^ purified B cells from three independent donors (D1, D2, and D3) were infected with EBNA3A-KO or EBNA3A-REV recombinant EBV and cultured for 30 days. RNA samples were taken at the times indicated after infection, and qPCR analysis was performed on each. STK39 mRNA expression was normalized to the endogenous control *GNB2L1*, and fold changes are shown relative to uninfected B cells at day 0. (B) EBNA3A, SPAK, and γ-tubulin protein expression during primary B cell infection with EBNA3A-KO or EBNA3A-REV recombinant EBV. (C) SPAK Western blot protein bands shown in panel B were analyzed by ImageJ software and represented based on the internal loading control γ-tubulin.

After establishing that *STK39* was robustly repressed by EBNA3A, we investigated what was happening early after infection of primary B cells with EBV. Using CD19^+^ peripheral B cells from independent donors, we reproducibly saw that infection with EBNA3A-REV virus (considered wild type [WT]) resulted in a rapid reduction of STK39 mRNA level (Fig. 4A). Around 15 days postinfection, STK39 mRNA was barely detectable. However, in the absence of EBNA3A (3A-KO), there was a failure to repress *STK39* that is consistent with the results derived from stable cell lines (Fig. 1). The same effect was seen at the protein level, where SPAK gradually decreased after infection with 3A-REV virus (Fig. 4B and C).

### ChIP-seq and ChIP-qPCR analysis reveal binding sites for both EBNA3A and EBNA3C at the *STK39* genomic locus.

**FIG 5:**
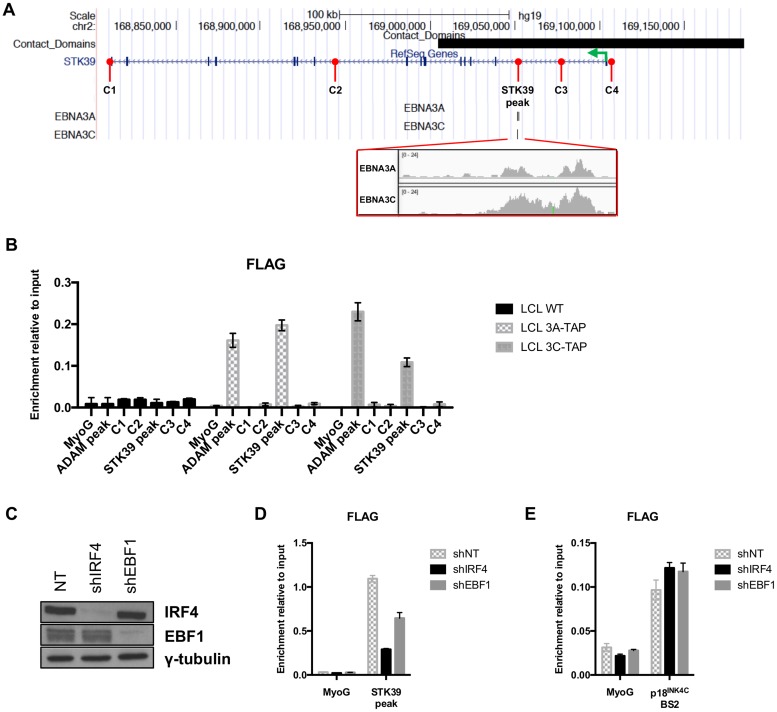
EBNA3A binds the *STK39* locus. (A) Schematic of the *STK39* genomic locus generated from the UCSC Genome Browser with the EBNA3A and EBNA3C peaks and contact domain (black rectangle). Sequencing reads are also shown around the EBNA3A and EBNA3C binding site (red rectangle). The green arrow shows the transcription start site of *STK39*. Positions of control primer (C1, C2, C3, and C4) pairs as well as the *STK39* peak primer pair used for qPCR to analyze precipitated DNA from ChIP are also shown in red. (B) ChIP qPCR analyses using anti-FLAG antibody to precipitate 3A-TAP or 3C-TAP and chromatin associated with it in LCL 3A-TAP or LCL 3C-TAP were performed. As a control for antibody specificity, a similar ChIP assay was performed using an LCL infected with wild type (B95.8-BAC; LCL WT). Primers for the Myoglobin promoter (MyoG) were used for qPCR as a negative control, whereas primers for known EBNA3A/3C binding sites at the *ADAM28/ADAMDEC1* intergenic enhancer (ADAM peak) were used as positive controls of EBNA3A/3C binding. Values represent ratios of chromatin precipitated, after correction for IgG, relative to 2.5% of input. Standard deviations are calculated from qPCR triplicates for each sample. (C) IRF4, EBF1, and γ-tubulin protein expression after infection of LCL 3A-TAP with lentiviruses carrying shRNA nontargeting (NT) control, shIRF4, or shEBF1 for 8 days. (D) LCL 3A-TAP infected with lentiviruses carrying a control nontargeting (NT) shRNA or shRNA directed against IRF4 (shIRF4) and EBF1 (shEBF1) for 8 days was subjected to ChIP qPCR analyses using anti-FLAG antibody as in panel B, to precipitate 3A-TAP and chromatin associated at the *STK39* peak. (E) Same as the experiment in panel D but using primers for the p18^INK4C^ site.

Interrogating a recent ChIP-seq analysis (14) performed on LCLs established with EBV recombinants expressing epitope-tagged EBNA3A (LCL 3A-TAP) or EBNA3C (LCL 3C-TAP) revealed a region—here called the *STK39* peak—about 53 kbp downstream of the transcription start site (TSS) of *STK39* (Fig. 5A). This peak includes two discrete binding sites for EBNA3A (300 nucleotides apart from each other, considered one binding site) and one binding site for EBNA3C that spans the two short EBNA3A sites. ChIP-qPCR confirmed a robust binding of EBNA3A-TAP and EBNA3C-TAP to the *STK39* peak (Fig. 5B). No binding was observed using control primer pairs covering four regions spanning the entire *STK39* locus. These data indicate that EBNA3A directly regulates *STK39* by binding to its locus.

Analysis of global chromatin looping data for the LCL (37) also revealed the presence of a contact domain containing the *STK39* peak and the *STK39* TSS (black rectangle, Fig. 5A). Contact domains are defined as regions with significant long-range association within them, suggesting that chromatin looping may be present in the LCL at the *STK39* locus between the EBNA3A binding site and the *STK39* TSS.

We were next interested in finding how EBNA3A was recruited to the *STK39* peak, as it cannot bind DNA directly. Interrogation of ENCODE project ChIP-seq data revealed that transcription factors IRF4 and EBF1 were both binding at the same site as EBNA3A on the *STK39* locus. Because these two transcription factors have been shown to colocalize with EBNA3A (13, 14), we assessed the role of these factors in the recruitment of EBNA3A on the *STK39* locus. Lentiviruses carrying a control nontargeting short hairpin RNA (shRNA) as well as shRNAs specific for IRF4 and EBF1 were produced and used to infect LCL 3A-TAP. The lentiviruses carrying shRNA against IRF4 and EBF1 efficiently depleted their target compared to the same cell line infected with lentiviruses expressing a nontargeting shRNA (Fig. 5C). Anti-FLAG ChIP assays were performed on these cells to assess the levels of EBNA3A-TAP bound to the *STK39* peak. Interestingly, we found a reduced level of EBNA3A on the *STK39* locus when either IRF4 or EBF1 was knocked down (Fig. 5D). No difference in EBNA3A binding was found on a control region on *CDKN2C* that did not contain any IRF4 or EBF1 binding sites (Fig. 5E). Taken together, those data demonstrate that the transcription factors IRF4 and EBF1 are important for the binding of EBNA3A onto the *STK39* genomic locus.

### EBNA3A increases the level of the repressive chromatin mark H3K27me3 at the *STK39* locus and recruits PRC2.

**FIG 6:**
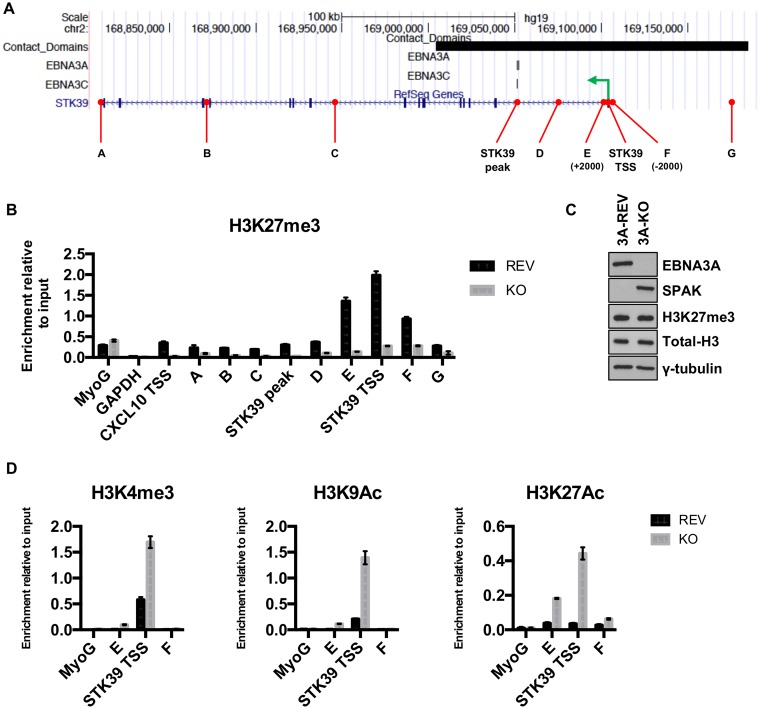
The *STK39* genomic locus is epigenetically modified by EBNA3A. (A) Schematic of the *STK39* genomic locus generated from the UCSC Genome Browser with the EBNA3A and EBNA3C peaks and contact domain (black rectangle). The green arrow shows the transcription start site of *STK39*. Positions of primer pairs used for qPCR to analyze precipitated DNA from ChIP are shown in red. (B) ChIP was performed on extracts from EBNA3A-KO and EBNA3A-REV LCLs (D3), and antibody specific for H3K27me3 was used. Primer pairs for Myoglobin (MyoG) and *GAPDH* were used as positive and negative controls, respectively, whereas a primer pair for the *CXCL10* TSS was used as a control for the cell lines. Values represent ratios of chromatin precipitated, after correction for IgG, relative to 2.5% of input. Standard deviations are calculated from qPCR triplicates for each sample. (C) EBNA3A, SPAK, H3K27me3, total H3, and γ-tubulin protein expression in LCL EBNA3A-KO or EBNA3A-REV carrying recombinant EBV used in panel B. (D) ChIP was performed on extracts from EBNA3A-KO and EBNA3A-REV LCLs used in panel B, and antibodies specific for H3K4me3, H3K9Ac, and H3K27Ac were used. Values represent ratios of chromatin precipitated, after correction for IgG, relative to 2.5% of input. Standard deviations are calculated from qPCR triplicates for each sample.

**FIG 7:**
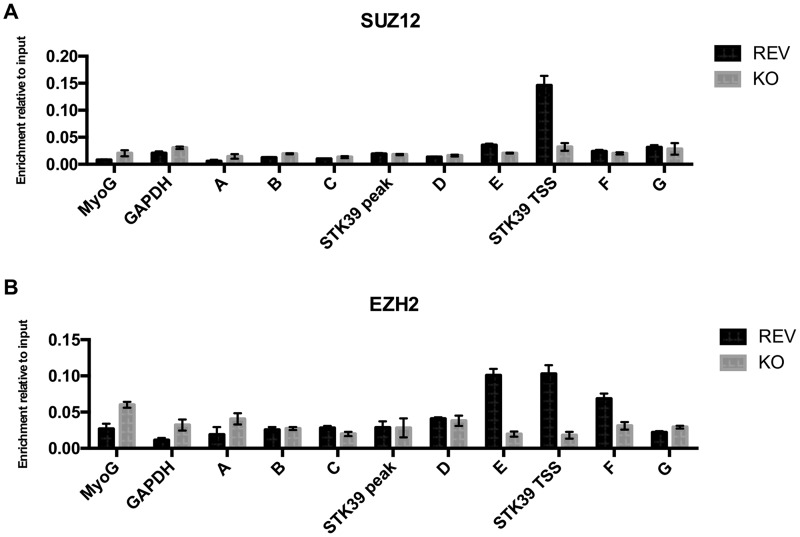
PRC2 is recruited to the *STK39* locus in LCL EBNA3A-REV. ChIP was performed on extracts from EBNA3A-KO and EBNA3A-REV LCL (D3), and antibodies specific for SUZ12 (A) and EZH2 (B) were used. Values represent ratios of chromatin precipitated, after correction for IgG, relative to 2.5% of input. Standard deviations are calculated from qPCR triplicates for each sample.

At several EBNA3A target genes, repression of transcription has been shown to correlate with the deposition of the histone H3 trimethyl lysine 27 (H3K27me3) silencing mark by the polycomb repressor complex 2 (PRC2) (31, 34). To determine whether the levels of H3K27me3 correlate with the silencing of the *STK39* gene by EBNA3A, ChIP analysis of EBNA3A-KO and EBNA3A-REV LCLs was performed using primers across the entire *STK39* genomic locus (Fig. 6A). When EBNA3A was expressed (3A-REV), H3K27me3 occupancy was significantly increased across the whole *STK39* locus, particularly around and at the transcription start site (TSS) (primer pair E-F), where the H3K27me3 level was high (Fig. 6B). Interestingly, the H3K27me3 level at the *STK39* TSS was four times higher than the level detected at the TSS of *CXCL10*, a well-known EBNA3A/3C-repressed gene (34, 35). This difference in H3K27me3 level detected by ChIP analysis between LCL 3A-REV and LCL 3A-KO was not due to a reduction of total H3K27me3 protein, as no difference was detected by Western blot analysis (Fig. 6C). Furthermore, we found that increased *STK39* transcription in LCL 3A-KO correlated with increased accumulation of activating histone marks H3K4me3, H3K9Ac, and H3K27Ac around the *STK39* transcription start site (Fig. 6D). Next, since trimethylation of H3K27 is catalyzed by PRC2, we explored whether this complex was recruited at the *STK39* genomic locus. ChIP analysis for PRC2 family members SUZ12 and EZH2 showed increased enrichment at the TSS of *STK39* in EBNA3A-REV (considered wild type) compared to EBNA3A-KO (Fig. 7A and B).

### PRC2 and the histone mark H3K27me3 are essential for the maintenance and establishment of *STK39* silencing by EBNA3A.

**FIG 8:**
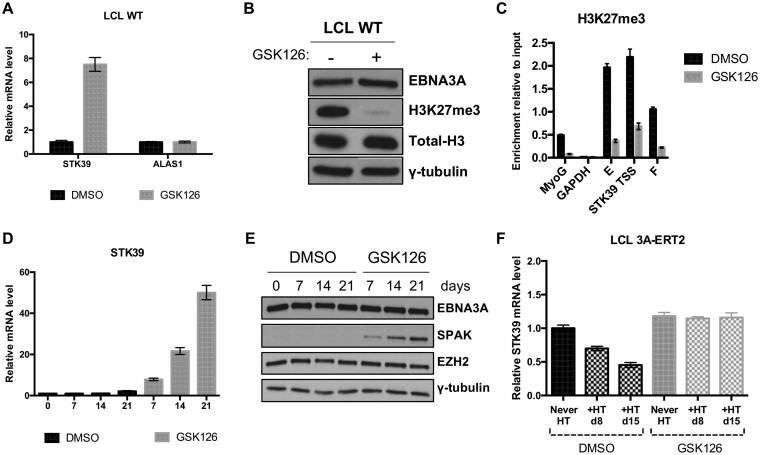
PRC2 and the histone modification H3K27me3 play an essential role in the EBNA3A-mediated repression of *STK39*. (A) An established wild-type (WT) (B95.8-BAC) LCL was treated with either the vehicle control DMSO or the EZH2 inhibitor GSK126 for 7 days. Analysis of expression of *STK39* and *ALAS1* was performed by qPCR, and mRNA expression was normalized to the endogenous control *GNB2L1* and is shown relative to each DMSO treatment. Standard deviations are calculated from qPCR triplicates for each sample. Data are representative of at least 3 independent experiments. (B) Western blotting extracts of the same cells as in the experiments in panel A show expression of EBNA3A, H3K27me3, total H3, and γ-tubulin. (C) H3K27me7 level was assessed by ChIP assay on cells used in the experiment in panel A. Values represent ratios of chromatin precipitated, after correction for IgG, relative to 2.5% of input. Standard deviations are calculated from qPCR triplicates for each sample. (D) Time course using WT LCL. Cells were grown over 21 days with either the vehicle control DMSO or GSK126. RNA samples were taken at the days indicated after infection, and qPCR analysis was performed on each. Gene expression for *STK39* was normalized to the endogenous control *GNB2L1* and is shown relative to day 0. Standard deviations are calculated from qPCR triplicates for each sample. (E) Western blotting extracts of the same cells used in the experiment in panel D show expression of EBNA3A, SPAK, EZH2, and γ-tubulin. (F) *STK39* mRNA expression using LCL 3A-ERT2 established without HT (Never HT). Cells were grown for 5 days with either the vehicle control DMSO or GSK126 before adding HT or not for 15 days. Gene expression for *STK39* was normalized to the endogenous control *GNB2L1* and is shown relative to the DMSO-treated 3A-ERT2 Never HT LCL. Standard deviations are calculated from qPCR triplicates for each sample.

We wanted to confirm that the histone mark H3K27me3 and therefore PRC2 were mechanistically involved in *STK39* silencing in LCLs. In order to do that, we used the compound GSK126, a specific inhibitor of EZH2 (38), the histone-lysine methyltransferase component of PRC2 responsible for the deposition of the H3K27me3 mark. The wild-type (WT) LCL was either mock treated (dimethyl sulfoxide [DMSO]) or treated with the inhibitor for 7 days and harvested for RNA and protein extraction. Interestingly, the level of STK39 mRNA greatly increased after EZH2 inhibition (Fig. 8A). This effect was specific, as analysis of control housekeeping gene *ALAS1* showed no change in the mRNA level after treatment. The GSK126 treatment was effective as it greatly reduced the total level of H3K27me3 in the LCL (Fig. 8B). The level of H3K27me3 around the *STK39* TSS was also strongly reduced after inhibition of EZH2 for 7 days (Fig. 8C). We then decided to follow the level of STK39 mRNA over a 3-week period after treatment with the EZH2 inhibitor, and we found that *STK39* increased over time at both the RNA and protein levels (Fig. 8D and E). Levels of EBNA3A and the methyltransferase EZH2 were not affected by the GSK126 treatment (Fig. 8E). Taken together, these data indicate that H3K27me3 is important for the maintenance of *STK39* silencing in LCLs.

Finally, we further characterized the role of PRC2 in *STK39* regulation. Deposition of H3K27me3 by PRC2 has been suggested to be the consequence rather than the cause of EBNA3A-mediated transcriptional shutdown of EBNA3A target genes (34). We tested whether H3K27me3 and therefore PRC2 were needed for the establishment of EBNA3A-mediated repression of *STK39*. To do that, we used an EBNA3A-conditional cell line that had been grown into an LCL without HT (designated Never HT [32]). We first treated the LCL 3A-ERT2 Never HT with either DMSO or GSK126 for 5 days in order to inhibit EZH2 before EBNA3A activation. Then, the cells were split and half of the culture was left without HT (Never HT) while HT was added to the other half (+HT) for 15 days. Samples of cells were harvested at 8 days and 15 days, and mRNA was extracted for analysis by reverse transcription (RT)-qPCR. Activation of EBNA3A, by the addition of HT, effectively repressed *STK39* expression in the DMSO mock-treated cells (Fig. 8F). However, the EBNA3A protein failed to silence *STK39* when EZH2 was inhibited (Fig. 8F), indicating that PRC2 is needed for the establishment of *STK39* silencing by EBNA3A.

### DNA methyltransferases are involved in *STK39* regulation in LCLs.

**FIG 9:**
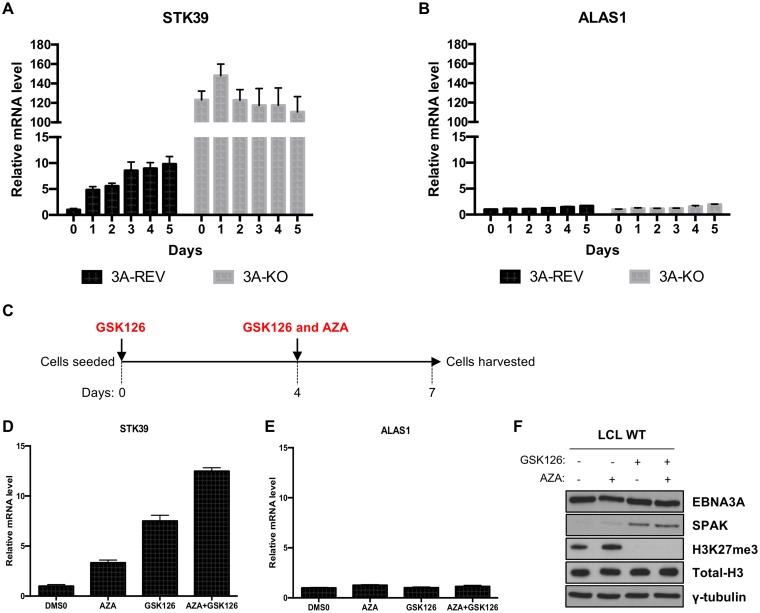
DNA methyltransferases are involved in the EBNA3A-mediated silencing of *STK39*. (A and B) LCL EBNA3A-REV and LCL EBNA3A-KO were treated with 5-azacytidine for 5 days. RNA samples were taken every day, and qPCR analysis was performed on each. *STK39* (A) and *ALAS1* (B) gene expression was normalized to the endogenous control *GNB2L1* and is shown relative to LCL 3A-REV at day 0. (C) Schematic of the experimental protocol of the dual drug treatment. WT LCLs were seeded at day 0 and treated with either DMSO or GSK126. At day 4, cells were split in half and treated with either DMSO, GSK126, DMSO with AZA, or GSK with AZA for 3 days before being harvested for analysis. (D and E) *STK39* (D) and *ALAS1* (E) gene expression was measured by qPCR, was normalized to the endogenous control *GNB2L1*, and is shown relative to each DMSO treatment at day 7. Standard deviations are calculated from qPCR triplicates for each sample. (F) Western blotting extracts of the same cells as used in the experiments in panels D and E show expression of EBNA3A, SPAK, H3K27me3, total H3, and γ-tubulin.

It has been demonstrated that the PRC2 family member EZH2 serves as a recruiting platform for DNA methyltransferases (39). Considering that EZH2 is involved in *STK39* regulation and considering that a recent study showed *STK39* to be repressed by DNA hypermethylation in B cell lymphoma (40), we assessed whether DNA methyltransferases were involved in the EBNA3A-mediated silencing of *STK39* in LCLs. EBNA3A-REV and EBNA3A-KO LCLs were treated with the DNA methyltransferase inhibitor 5-azacytidine (AZA) for 5 days (Fig. 9). Samples of cells were harvested each day, and mRNA was extracted for analysis by reverse transcription (RT)-qPCR. In the LCL expressing EBNA3A, where *STK39* is silenced, treatment with AZA led to a gradual increase of STK39 mRNA over time, reaching a plateau after 3 days about 8-fold higher than the starting level (Fig. 9A). In contrast, in an EBNA3A-negative LCL (3A-KO) where *STK39* is derepressed, STK39 mRNA was not affected by the DNA methyltransferase inhibitor treatment (Fig. 9A). Relative expression of the control gene *ALAS1* showed no regulation after AZA treatment in both cell lines (Fig. 9B). As *STK39* was recently shown to be silenced in B cell lymphoma through DNA methylation around the *STK39* transcription start site (40), we attempted to assess the DNA methylation status of the CpG island around the *STK39* TSS in LCLs. However, we could not see a difference in DNA methylation between LCL 3A-REV and LCL 3A-KO around the *STK39* TSS region (data not shown). Furthermore, we not only found that the methylation pattern was the same but we also found that the *STK39* TSS region was rather unmethylated in LCL.

As we found that STK39 mRNA was derepressed in the LCL after treatment with an EZH2 inhibitor (GSK126) or a DNA methyltransferase inhibitor (AZA), we tested whether a combined treatment would lead to a further increase of *STK39*. To do that, we performed an experiment where WT LCLs were treated with the EZH2 inhibitor for 7 days (Fig. 9C). At the 4th day, cells were split and either mock treated or treated with AZA for 3 days. After a week, the cells were harvested for mRNA and protein extraction. As expected, treatment with either GSK126 or AZA led to an increase of STK39 mRNA (Fig. 9D). Interestingly, a combined treatment led to a further increase compared to single treatments. Relative expression of the control housekeeping gene *ALAS1* was not affected by any treatment (Fig. 9E). Some cooperation, though less pronounced, was also seen at the SPAK protein level after the combined treatment (Fig. 9F).

## DISCUSSION

In this study, we have analyzed the molecular mechanisms by which the Epstein-Barr virus protein EBNA3A silences *STK39*. Using LCLs carrying knockout, revertant, or conditional-EBV recombinant, we explored the role of each of the EBNA3 proteins in the regulation of *STK39* and found that only EBNA3A is needed for this function. This is the first time that the EBNA3A protein has been found to be the only EBV transcriptional regulator of a cellular gene. *STK39* was quickly silenced after infection of primary B cells by EBV. Furthermore, this repression was shown to be reversible, as inactivation of EBNA3A in LCL 3A-ERT2 induced the reexpression of *STK39*. EBNA3A was then shown by ChIP experiments to bind a genomic region about 53 kbp upstream of the *STK39* coding sequence. Since this region is far away from the *STK39* TSS, we assume that the EBNA3A repression may be mediated through long-range chromatin looping. Long-range chromatin looping regulation has been reported for almost all genes regulated by the EBNA3 proteins (30, 32, 33, 36). Even though we have not formally tested this hypothesis, global chromatin looping data available on LCLs revealed a large contact domain between the EBNA3A binding site and the *STK39* TSS, suggesting regulation by chromatin looping.

EBNA3C was found to bind to the same region as EBNA3A on the *STK39* genomic locus even though it had no role in *STK39* expression. This likely reflects the ability of EBNA3A and EBNA3C to interact with each other (25). The reciprocal phenomenon has recently been demonstrated at another gene: EBNA3A binds on the same site as EBNA3C on *COBLL1*, but the gene is regulated by only EBNA3C (11). The presence of EBNA3C at the *STK39* locus is likely to be due to its capacity to bind to EBNA3A, but at this stage, we cannot rule out the possibility that EBNA3A and EBNA3C both independently bind to the *STK39* genomic locus. It is well known that EBNA3 cannot bind DNA directly and binds gene control elements through interaction with cellular transcription factors. Previous studies found that EBNA3 binding sites coincided with many cellular transcription factors such as CBFβ, RUNX3, BATF, IRF4, and EBF1 (12–16). Interestingly, ChIP-seq data from the ENCODE project for various transcription factors in the GM12878 LCL revealed that the *STK39* peak was bound by IRF4 and EBF1. Depletion of both IRF4 and EBF1 led to a significant reduction of EBNA3A recruitment to the STK39 peak, indicating that both IRF4 and EBF1 are important for EBNA3A binding at the *STK39* genomic locus. The IRF4 transcription factor has been shown to extensively colocalize with the EBNA3A binding site (13, 14), and it has been suggested that it might assemble with EBNA3A through high-order complexes with the B cell transcription factor BATF (13). EBNA3A ChIP-seq has demonstrated that EBNA3A binding sites are less enriched for EBF1 than EBNA3C or EBNA3B binding sites (13). The cellular factor EBF1 has been shown to interact with the EBV transcription factor EBNA2 and recruits the viral protein to a specific genomic location (41, 42). However, EBNA2 does not seem to be involved in the STK39 mRNA regulation (43, 44), and no EBNA2 binding site has been found at *STK39* in EBNA2 ChIP-seq studies (33). The fact that depletion of EBF1 decreased the EBNA3A binding to the STK39 genomic locus is interesting as EBF1 has, so far, not been shown to be involved in specific EBNA3A-mediated transcriptional regulation. Finally, it is important to note that, at this stage, we cannot rule out the possibility that depletion of either IRF4 or EBF1 might change the conformation of transcription factor complexes (including IRF4 or EBF1) at the *STK39* peak, leading to a reduction in EBNA3A recruitment.

Since EBNA3A-mediated repression has been shown to involve recruitment of PRC2 to target genes and the deposition of H3K27me3, we investigated if this mechanism was used by EBNA3A to silence *STK39*. The level of this epigenetic repressive mark was found to be highly elevated in LCLs expressing EBNA3A compared to EBNA3A-deficient LCLs and was spread throughout the *STK39* locus with a peak around the *STK39* TSS. The H3K27me3 level around the *STK39* TSS was found to be even higher than our positive control at the *MyoG* gene. Interestingly, this very high level of H3K27me3 was also seen at *COBLL1* (a gene regulated by only EBNA3C) but not at the *ADAM28*/*ADAMDEC1* locus coregulated by EBNA3A and -3C (11). It is tempting to speculate that the very high level of the repressive mark H3K27me3 might be a feature of genes regulated by either only EBNA3A or only EBNA3C. H3K27me3 being catalyzed by PRC2, we investigated the levels of PRC2 members SUZ12 and EZH2 at the *STK39* locus. We found an elevated level of SUZ12 and EZH2 in LCL 3A-REV around the *STK39* TSS which correlated with the H3K27me3 level. In a previous study on EBNA3A target genes *CXCL9* and *CXCL10* (34), Harth-Hertle and colleagues showed that SUZ12 and EZH2 levels were both elevated within the entire chromatin domain encoding CXCL9 and CXCL10 when EBNA3A was expressed. This is not what we saw at the *STK39* locus, suggesting that the molecular mechanism behind *STK39* silencing by EBNA3A is different. Interestingly, EBNA3A-mediated silencing of *STK39* could be reversed by the inhibition of EZH2, which reduced global H3K27me3 level in LCLs, suggesting that H3K27me3 epigenetic modification (and hence PRC2) is important for the maintenance of STK39 silencing.

It is currently unclear whether PRC2 is recruited by direct interaction with EBNA3A or if this represents a default mechanism of gene repression.

PRC2 recruitment and deposition of the repressive mark H3K27me3 to EBNA3A target genes were indeed thought to be a consequence of the initial establishment of EBNA3A-mediated repression and therefore were important only for the maintenance of gene repression (34). In this study, we demonstrated that without a functional PRC2, EBNA3A was unable to induce STK39 silencing. This clearly demonstrates that H3K27me3 and therefore PRC2 not only are important for the maintenance of *STK39* repression in LCLs but are actually needed for the initial establishment of *STK39* silencing by EBNA3A.

Epigenetic alterations that silence gene expression play an important role in cancer (45). The polycomb repressor system is an epigenetic mechanism that plays a major role in the maintenance of the transcriptional state initially established by transcription factors. In the last few years, it has emerged that the polycomb proteins and the repressive mark H3K27me3 are closely linked to DNA methylation, another epigenetic mechanism involved in gene regulation (18, 39, 46). EBNA3A and EBNA3C have been shown to silence *BCL2L11* with concomitant recruitment of PRC2, leading to an increased level of H3K27me3 while DNA methylation at the BCL2L11 promoter CpG island was found in EBV-positive BL cells (25, 47, 48). In this study, we showed that inhibition of DNA methyltransferase (AZA treatment) induced the reexpression of *STK39* only in LCLs that expressed EBNA3A. Interestingly, it was recently shown that *STK39* is silenced in B cell lymphoma through DNA hypermethylation around the *STK39* transcription start site (40). However, we could not find DNA methylation around the *STK39* TSS in LCLs. Analysis of DNA methylation data from the ENCODE project confirmed that the *STK39* TSS in LCLs was unmethylated and revealed no DNA methylation around the *STK39* peak. This suggests that DNA methylation is not involved in the regulation of the *STK39* genomic locus in LCLs. The drastic effect seen on *STK39* expression after 5-azacytidine treatment is likely to be DNA methylation independent. Indeed, DNA methyltransferases have been shown to repress transcription in a DNA methylation-independent manner (49, 59–61), and it has been shown that genes silenced by H3K27me3 can also be DNA methylation independent (50). Finally, it is worth noting that, at this stage, we cannot exclude the possibility of an indirect effect of the 5-azacytidine treatment leading to the derepression of a potent *STK39* transcriptional activator. Our data demonstrated that treatment with both AZA and EZH2 inhibitors had a cumulative effect on *STK39* expression compared with independent treatments, demonstrating that these modifications are independent and have a distinct or additive effect on the *STK39* repression. However, we cannot conclude whether PRC2 is involved in the recruitment of DNMTs to the *STK39* locus.

The *serine/threonine kinase 39* gene (*STK39*) encodes the STE20 (sterile 20-like)-related proline-alanine-rich kinase (SPAK), one of two members of the germinal center kinase VI subgroup within the STE20 kinase family (51). SPAK is a 60-kDa kinase protein well known for its role in the regulation of cellular ion homeostasis through direct interaction and phosphorylation of cation chloride cotransporters. SPAK has been shown to be involved in various biological activities, such as cell differentiation (51–53), transformation (51, 54), autophagy (55), and cytoskeleton rearrangement and cell migration (56, 57). Interestingly, *STK39* has been shown to be well expressed in naive, germinal center, and memory B cells, but its expression is silenced in B cell lymphomas, leading to the loss of SPAK expression (40). In the same study, this silencing was also shown to protect B cells from caspase-dependent apoptosis induced by DNA double-strand breaks. The biological relevance of *STK39* silencing by EBNA3A is unknown and is under investigation. Considering the *STK39* role in B cell lymphoma, it is tempting to speculate that silencing of *STK39* in primary B cells by EBV might be an important step for the suppression of apoptosis induced after infection.

In conclusion, we described the silencing of the cellular gene *STK39* by the Epstein-Barr virus protein EBNA3A. Our results revealed that, for the first time, EBNA3A does not need any other EBNA3 members for its transcription repression function at this gene. EBNA3A directly regulates *STK39* by binding to its genomic locus and initiates its repression through recruitment of the PRC2 with deposition of H3K27me3. Furthermore, we provided evidence of the involvement of DNA methyltransferases in this repression. These findings will help our understanding of gene regulation by EBV transcription factors that leads to the reprogramming of cellular gene expression, which can drive lymphoma development.

## MATERIALS AND METHODS

### Ethics statement.

The buffy coat residues used in this study for the isolation of CD19^+^ primary B cells were purchased from the UK Blood Transfusion service. As these were derived from anonymous volunteer blood donors, no ethical approval is required.

### Microarray analysis.

RNA from two independent 3A-ERT2 LCLs cultured with or without HT for 30 days was extracted using the RNeasy minikit from Qiagen and hybridized to Affymetrix Human Exon 1.0ST microarrays by UCL Genomics. Gene-level analysis was performed using the MMBGX algorithm to generate gene-level data according to ENSEMBL genome annotation version 64, as mapped to the Exon microarray by AnnMap, broadly as described previously (58).

### Cell culture.

Cells were cultured at 10% CO_2_ and 37°C in RPMI 1640 medium supplemented with 10% fetal calf serum (FCS), penicillin, and streptomycin. Cells were routinely seeded at 0.3 × 10^6^ cells/ml 1 day before harvesting. The activating ligand 4-hydroxytamoxifen (HT) was added at 400 nM, EZH2 inhibitor (GSK126) was added at 4 μM, and 5-azacytidine was added at 5 μM, where indicated. These supplements were added to cultures every time that fresh medium was added to the cells. Where indicated, cells grown in culture medium containing HT were washed three times in fresh medium and resuspended in fresh medium with HT omitted (noted as “washed” cell populations).

### Production of lentiviruses.

shRNA for the nontargeted control (target sequence 5′-CCTAAGGTTAAGTCGCCCTCG-3′), IRF4 (target sequence 5′-CCGCCATTCCTCTATTCAAGA-3′), and EBF1 (target sequence 5′-GCAGTCTCTGATAACATGTTT-3′) was constructed in pLKO1 plasmid as described previously (14). For viral packaging, lentivirus-based vectors were cotransfected with helper plasmids psPAX2 and pMD2.G into 293T cells by the calcium phosphate precipitation method. Medium containing the lentivirus was collected 48 h after transfection.

### Viral infection of cells.

Infection of fresh primary B cells and time course experiments were performed as described previously (31). For lentiviral infections of LCLs, 20 × 10^6^ cells were pelleted and resuspended in 1 ml of lentivirus-containing 293T medium with 8 μg/μl Polybrene. Cells were then centrifuged at 450 × *g* for 1.5 h at room temperature. The cells were then resuspended in 5 ml of complete RPMI medium and transferred to flasks. Two days later, a further 5 ml was added, containing puromycin to a final concentration of 1 μg/ml.

### qPCR.

RNA was isolated from cells using an RNeasy minikit (Qiagen) with DNase digestion as in the manufacturer's instructions. Reverse transcription of RNA into cDNA was performed using Superscript III First Strand Synthesis Supermix for reverse transcription-quantitative PCR (qRT-PCR) (Invitrogen). Ten nanograms of cDNA was used per quantitative PCR (qPCR) mixture using the Platinum SYBR green qPCR Supermix uracil DNA glycosylase (UDG) kit (Invitrogen), and PCR was performed on an ABI 7900HT real-time PCR machine. The PCR primers used in the study were as follows: STK39, 5′-CTCTGTGCACGACTCTCAGG-3′ and 5′-GAGCAAACCCAATCAGCTTC-3′; ALAS1, 5′-TCCACTGCAGCAGTACACTACCA-3′ and 5′-ACGGAAGCTGTGTGCCATCT-3′; GNB2L1, 5′-GCTTGCAGTTAGCCAGGTTC-3′ and 5′-GAGTGTGGCCTTCTCCTCTG-3′; BFL-1, 5′-TTACAGGCTGGCTCAGGACT-3′ and 5′-AGCACTCTGGACGTTTTGCT-3′; pri-miR-221/222, 5′-ACTTGCCCTCCTTTCCTTTC-3′ and 5′-AGGTGTTTCCGACGCATTAC-3′; CXCL9, 5′-GTAGTGAGAAAGGGTCGCTGT-3′ and 5′-AGGGCTTGGGGCAAATTGTT-3′; ADAM28, 5′-GTTGCAGGGACAATGGCACA-3′ and 5′-TGAGACGGCTGCAGGAACTG-3′; COBLL1, 5′-CTGTTCAGCTGACAACAGATCG-3′ and 5′-ACGTTGAACTCTCAGTGGTCCT-3′. The comparative threshold cycle (ΔΔ*C_T_*) method was used to calculate relative mRNA expression with the housekeeping gene *GNB2L1* used as an endogenous control. Dissociation curve analysis was performed during each run to confirm the absence of nonspecific products. Error bars in graphs are the standard deviation for three triplicate qPCR replicates for each mRNA sample.

### Immunoblotting.

SDS-polyacrylamide gel electrophoresis and Western blotting were performed as described previously (26). Antibodies used for Western blotting were those to EBNA3A (Abcam; catalog no. ab16126), EBNA3B (Allday lab; clone 6C9), EBNA3C (kind gift from M. Row, University of Birmingham; clone A10), γ-tubulin (Sigma; catalog no. T6557), SPAK (Cell Signaling; catalog no. 2281), H3K27me3 (Millipore; catalog no. 07-449), total H3 (Abcam; catalog no. ab1791), EZH2 (Cell Signaling; catalog no. 5246), IRF4 (Millipore; catalog no. 04-112), and EBF1 (Millipore; catalog no. AB10523). In all blotting assays, γ-tubulin was used as a loading control.

### ChIP.

Chromatin immunoprecipitation (ChIP) assay and qPCR analysis were performed as described previously (11). Sequences of the primers used in these assays are listed in Table 2. Antibodies used for ChIP experiments were those to FLAG (Cell Signaling; catalog no. 2368), H3K27me3 (Millipore; catalog no. 07-449), H3K4me3 (Millipore; catalog no. 17-614), H3K9Ac (Millipore; catalog no. 17-658), H3K27Ac (Millipore; catalog no. 17-683), SUZ12 (Abcam; catalog no. ab12073), and EZH2 (Cell Signaling; catalog no. 5246). All ChIPs shown are representative of at least 2 independent experiments.

**TABLE 2:** List of primers used to analyze precipitated DNA from ChIP

Target	Primer name	Sequence (5′→3′)
STK39	C1 or A	F-GTCCTGGTAAGACAAATAGCAC
		R-AACCTCGACTCTGCTCTGGA
	B	F-CTAGACGGGGGTTTGACAGA
		R-TTAGGGCAGCTTTTGGTGTC
	C2 or C	F-GCCACAGTTTTGTGCCTGAT
		R-GGGAGAATGTTGCTTTTCCA
	STK39 peak	F-TCACTTTTACATCCTCGTTGTCAAG
		R-TTTGTTGCCCAGGTTATGTC
	C3 or D	F-CGCAACAGCAACAAATGCCC
		R-GGTTTTCTCTTTGCCCCAGG
	E	F-TCAACTGATATGCACTTTTCCCGG
		R-ATTTTAAAGGCACCCGCCTC
	STK39 TSS	F-CAGCACCAACGCTCATAGAA
		R-GGCCTACTGGCTTCAACAGT
	C4 or F	F-TGCTAGCAAGTGGGGCAATGC
		R-TGCCGTGGTTTCTTCTGCCC
	G	F-CAGGCTTCCCAAACACAAAT
		R-TTTCTGAAACGGCTCTCACC
Myoglobin	MyoG	F-GGAGAAAGAAGGGGAATCACA
		R-GATAAATATAGCCAACGCCACA
GAPDH^*a*^	GAPDH	F-CGCTCTCTGCTCCTCC
		R-TTTCTCTCCGCCCGTCCAC
ADAM gene cluster	ADAM peak	F-CTTCATGGCTACAGACTCTTGG
		R-CCTATGTCTCGCTTCCTGCT
CXCL10	CXCL10 TSS	F-TCCCTCCCTAATTCTGATTGG
		R-AGCAGAGGGAAATTCCGTAAC

a GAPDH, glyceraldehyde-3-phosphate dehydrogenase.
